# Discrimination of breast cancer from benign tumours using Raman spectroscopy

**DOI:** 10.1371/journal.pone.0212376

**Published:** 2019-02-14

**Authors:** Fiona M. Lyng, Damien Traynor, Thi Nguyet Que Nguyen, Aidan D. Meade, Fazle Rakib, Rafif Al-Saady, Erik Goormaghtigh, Khalid Al-Saad, Mohamed H. Ali

**Affiliations:** 1 Centre for Radiation and Environmental Science, FOCAS Research Institute, Technological University Dublin, Dublin, Ireland; 2 Department of Chemistry and Earth Sciences, Qatar University, Doha, Qatar; 3 Pathology and Laboratory Medicine, Al Ahli Hospital, Doha, Qatar; 4 Center for Structural Biology and Bioinformatics, Laboratory for the Structure and Function of Biological Membranes, Université Libre de Bruxelles, Brussels, Belgium; 5 Qatar Biomedical Research Institute, Doha, Qatar; University of South Alabama Mitchell Cancer Institute, UNITED STATES

## Abstract

Breast cancer is the most common cancer among women worldwide, with an estimated 1.7 million cases and 522,000 deaths in 2012. Breast cancer is diagnosed by histopathological examination of breast biopsy material but this is subjective and relies on morphological changes in the tissue. Raman spectroscopy uses incident radiation to induce vibrations in the molecules of a sample and the scattered radiation can be used to characterise the sample. This technique is rapid and non-destructive and is sensitive to subtle biochemical changes occurring at the molecular level. This allows spectral variations corresponding to disease onset to be detected. The aim of this work was to use Raman spectroscopy to discriminate between benign lesions (fibrocystic, fibroadenoma, intraductal papilloma) and cancer (invasive ductal carcinoma and lobular carcinoma) using formalin fixed paraffin preserved (FFPP) tissue. Haematoxylin and Eosin stained sections from the patient biopsies were marked by a pathologist. Raman maps were recorded from parallel unstained tissue sections. Immunohistochemical staining for estrogen receptor (ER) and human epidermal growth factor receptor 2 *(*HER2/neu) was performed on a further set of parallel sections. Both benign and cancer cases were positive for ER while only the cancer cases were positive for HER2. Significant spectral differences were observed between the benign and cancer cases and the benign cases could be differentiated from the cancer cases with good sensitivity and specificity. This study has shown the potential of Raman spectroscopy as an aid to histopathological diagnosis of breast cancer, in particular in the discrimination between benign and malignant tumours.

## Introduction

Breast cancer is the second most common cancer worldwide after lung cancer and the most common cancer among women with approx. 1.7 million new cancer cases diagnosed in 2012 and 522,000 deaths [1].

For breast cancer, as for most cancers, the gold standard diagnostic technique is biopsy followed by histopathology, where the excised tissue is processed, cut into sections and mounted on a glass slide for examination by a pathologist. It is widely acknowledged that histopathology is subjective as it relies mainly on morphological information resulting in inter-observer disagreement [2]. Visual assessment of tissue architecture and individual cells is employed to grade tumours but the grading criteria can be subjective and pre-cancer changes may not be visually apparent. In addition, because of the structural complexity and heterogeneous nature of breast tissue, it can be difficult for histopathologists to find and classify abnormal areas of tissue [3].

In addition to standard histopathology, a limited number of biomarkers can be used to support the diagnosis. In breast cancer, analysis of oestrogen and progesterone receptors by immunohistochemistry can help identify those patients that are likely to respond to hormone treatment [4]. In addition, expression of human epidermal growth factor receptor 2 (HER2) identifies patients who will respond to herceptin (trastuzumab), a drug which reduces the risk of recurrence and mortality in patients with HER2 positive early stage breast cancer [5].

Vibrational spectroscopy techniques, such as Raman spectroscopy, have recently shown great potential for disease diagnosis [6–8]. Raman spectroscopy is based on inelastic light scattering. Monochromatic laser light is used to illuminate the sample and scattering of the light occurs due to interactions between the incident photons and the molecules in the sample. The energy of this inelastically scattered light is reduced by an amount equal to the vibrational energy of the molecules in the sample. Thus, in depth information on the molecular composition of a sample can be obtained from the positions, relative intensities and shapes of the Raman bands. Thus, Raman spectroscopy can provide a biochemical fingerprint of a tissue biopsy and, together with advanced data analysis techniques, have been shown to classify normal, benign, pre-cancer and cancer cases.

Early studies on breast tissues showed that Raman spectra of diseased breast tissue (benign and malignant) showed reduced lipids and carotenoids compared to normal breast tissue [9–12]. A number of later studies by Abramczyk and colleagues similarly showed that the main differences between normal and cancer tissues were in spectral regions associated with vibrations of carotenoids, fatty acids and proteins [13–15]. In particular, unsaturated fatty acids were found to be important for differentiation of normal and cancerous breast tissues [16]. Significant changes in Raman spectral bands associated with carotenoids and lipids have also been reported in breast cancer tissue following chemotherapy [17].

Feld and co-workers showed that Raman spectroscopy could discriminate infiltrating carcinoma from normal and benign tissues *ex vivo* with a sensitivity of 94% and specificity of 96% [18]. Generally, a higher fat content was observed for normal tissues compared to a higher collagen content in all abnormal breast tissues. A follow up prospective study on freshly excised surgical specimens validated the previously developed algorithm [18] and showed that Raman spectroscopy could discriminate cancer tissue from normal and benign tissues with a sensitivity of 83% and a specificity of 93% [19]. Similarly, Kong et al showed that normal breast tissue could be discriminated from ductal carcinoma with 95.6% sensitivity and 96.2% specificity based on increased concentration of nucleic acids and reduced concentration of collagen and fat in the cancer tissue [20].

The aim of the present study was to discriminate between benign lesions (fibrocystic, fibroadenoma, intraductal papilloma) and cancer (invasive ductal carcinoma and lobular carcinoma) using formalin fixed paraffin preserved (FFPP) tissue.

## Materials and methods

### Sample collection and processing

Formalin fixed paraffin preserved (FFPP) breast tissue representing benign tumors, fibrocystic lesions, fibroadenoma and intraductal papilloma, and cancers, invasive ductal carcinoma and lobular carcinoma, were cut into 10 micron sections and mounted on glass slides. Tissue samples were collected from twenty individual patients and multiple sections were collected from each sample to be measured and analyzed. Four parallel sections were prepared, one unstained section for Raman spectroscopy, one for routine histology (Haematoxylin and Eosin (H & E) staining) and the other two sections for immunohistochemistry.

Although a previous study has shown biochemical changes due to sample processing [21], FFPP breast tissue sections were de-paraffinized using xylene and further rehydrated through graded alcohols to distilled water following standard laboratory procedures. The pathologist marked the regions of interest on the stained (H & E) sections. These breast tissue samples were obtained from Al-Ahli Hospital with an ethical approval (dated on 17.01.2018), Doha-Qatar. The samples were analyzed by Dublin Institute of Technology after obtained bioethical approval (Ref 13–28), Ireland. All experimental protocols were approved by the collaborative institutues and adhere with (to) the relevant guidelines and regulations. All the material was taken anonymously (as appeared in the ethical approval) and a consent form from Al-Ahli Hospital was signed by all patients undergoing any procedure.

### Immunohistochemistry

Two further parallel sections from each tissue block were used for IHC. After blocking endogenous peroxidase activity with 3% hydrogen peroxide in methanol, antigen retrieval was achieved by heating the slides in 10 mmol/l citrate buffer (pH 6) using a water bath. Primary antibodies to HER2/neu and ER were applied and the Avidin-Biotin peroxidase (ABC) kit (Vectastain) was used for application of the secondary antibody. Signals were developed with Diaminobenzidine (DAB) followed by light nuclear counter staining with Mayer’s Haematoxylin. Each set of slides was run with a known positive and negative control.

### Raman spectroscopy

Raman spectroscopy was performed using a Horiba Jobin Yvon Labram HR800 UV system, which was equipped with a 532 nm solid-state diode laser that delivered 100 mW of power to the sample. Spectral maps were recorded from regions of the sample containing clinically significant morphological changes associated with each condition, which were marked by a clinical pathologist on the parallel H & E stained section. The laser excitation was delivered to the sample through a x100 objective lens and the spectra were dispersed onto the detector using a diffraction grating ruled with 1200 lines/mm providing a spectral resolution of 3 cm^-1^ per pixel. The confocal hole was set to 100 μm such that contributions from the glass were minimised. The system was calibrated daily using the 520.7 cm^-1^ line of silicon. Each individual tissue spectrum was measured with a 10 second integration time averaged over 3 successive measurements.

#### Data pre-processing

All spectral processing procedures were conducted using Matlab (R2017a; Mathworks Inc., Natick, MA), along with in-house developed algorithms and procedures available within the PLS Toolbox (v 8.0.2, Eigenvector Research Inc., Wenatchee, MA). Briefly, spectra were imported, baseline was subtracted with a rubberband algorithm, and those spectra whose SNR deviated by more than 30% from the mean in their sample were discarded. Spectra were then vector normalised. Finally all spectra were smoothed using a Savitzky-Golay smoothing algorithm with a 7-point window and a 5^th^ order polynomial.

#### Data analysis

Principal component analysis (PCA) was conducted to determine whether spectra could be differentiated with respect to their overall class Benign and Cancer. Principal Components (PCs) were generated and subjected to linear discriminant analysis (LDA), quadratic discriminant analysis (QDA) and a series of Support Vector Machine (SVM) classifiers, Linear c-SVC, Linear nu-SVC, RBF c-SVC and RBF nu-SVC. Each classifier was applied on 50 datasets for PCA-LDA and PCA-QDA and 20 datasets for SVM composed of PC scores with increasing numbers of PCs. Partial least squares discriminant analysis (PLSDA) was also applied on 50 datasets composed of increasing numbers of latent variables (LVs). For each application, the classifier was trained using 60% of the spectra randomly selected from all spectra, and tested using the remaining held out 40%. PLSDA was also applied with leave one patient out cross validation (LOPOCV).

## Results

**Fig 1:**
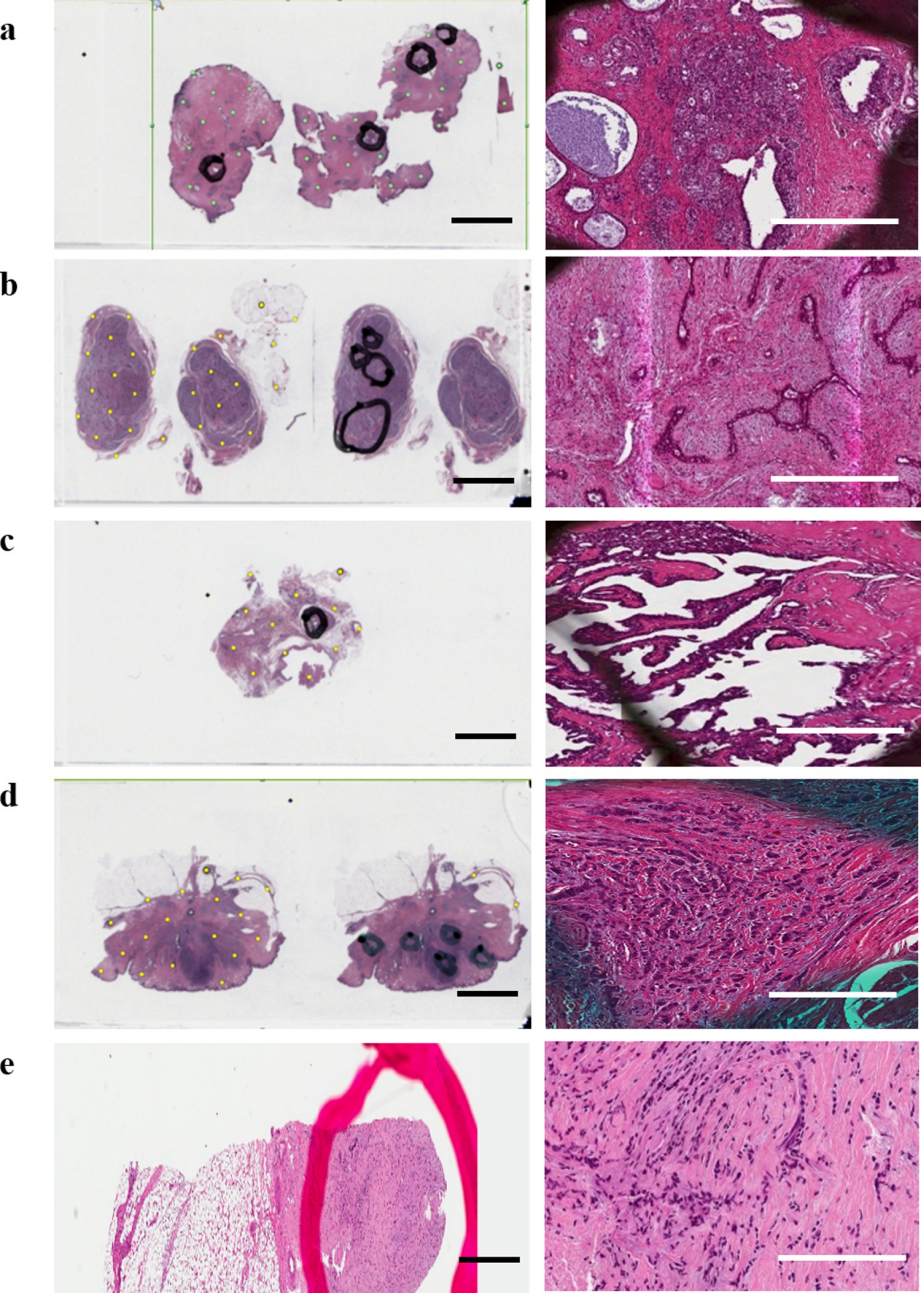
**Digital pathology scan of H & E stained tissue section (left) and magnified image of one of the regions of interest marked by the pathologist (right) for (a) fibrocystic lesion, (b) fibroadenoma, (c) intraductal papilloma, (d) infiltrative ductal carcinoma, (e) infiltrative lobular carcinoma.** H&E (scale bar 2.5 cm) & Magnified images (scale bar 0.1mm).

Fig 1 shows H&E stained tissue sections from each benign lesion and cancer type. A fibrocystic lesion showing dilated ducts and cyst formation with sclerosing adenosis is depicted in Fig 1A, while Fig 1B depicts fibroadenoma showing both epithelial and stromal components and Fig 1C depicts intraductal papilloma showing dilated ducts with papillary projections having a fibrovascular core and epithelial-myoepithelial lining. Infiltrative ductal carcinoma is depicted in Fig 1D which shows proliferating neoplastic cells, some forming tubules with moderate desmoplastic reaction and mild lymphocytic infiltration, and infiltrative lobular carcinoma is depicted in Fig 1E which shows tumour cells infiltrating singly (with no tendency for tubule formation) in a linear pattern and loosely dispersed in a fibrous matrix.

**Fig 2:**
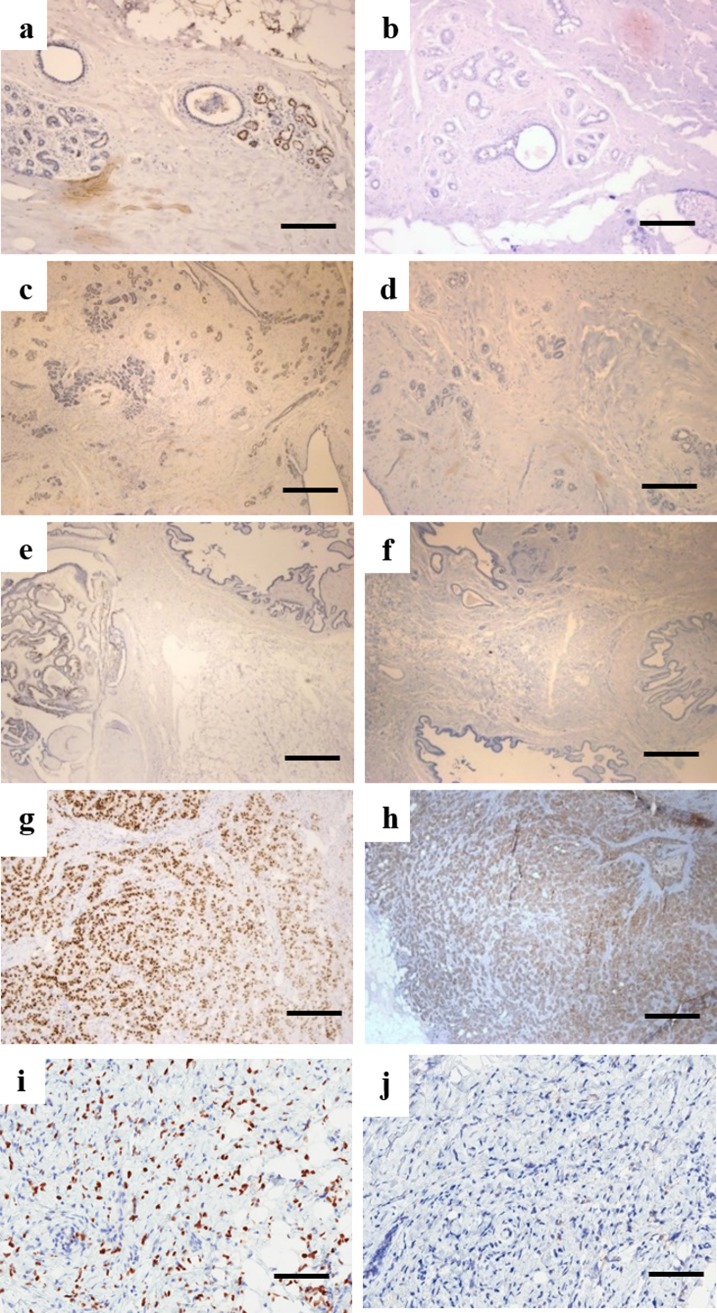
**Representative images for ER immunostaining for (a) fibrocystic lesion, (c) fibroadenoma, (e) intraductal papilloma, (g) infiltrative ductal carcinoma, (i) infiltrative lobular carcinoma and HER2/neu immunohistochemistry staining for (b) fibrocystic lesion, (d) fibroadenoma, (f) intraductal papilloma, (h) infiltrative ductal carcinoma, (j) infiltrative lobular carcinoma (Scale bar 0.2 mm)**.

Immunohistochemical staining of ER and HER2/neu in each benign lesion and cancer type are shown in Fig 2. Patchy positivity for ER was observed in fibrocystic disease (Fig 2A), fibroadenoma (Fig 2C) and intraductal papilloma (Fig 2E), while expression of HER2/neu was negative in fibrocystic disease (Fig 2B), fibroadenoma (Fig 2C) and intraductal papilloma (Fig 2E). ER was observed to be strongly and diffusely positive in both infiltrative ductal carcinoma (Fig 2G) and infiltrative lobular carcinoma (Fig 2I). HER2/neu was observed to be positive in infiltrative ductal carcinoma (Fig 2H) and negative in infiltrative lobular carcinoma (Fig 2J).

**Fig 3:**
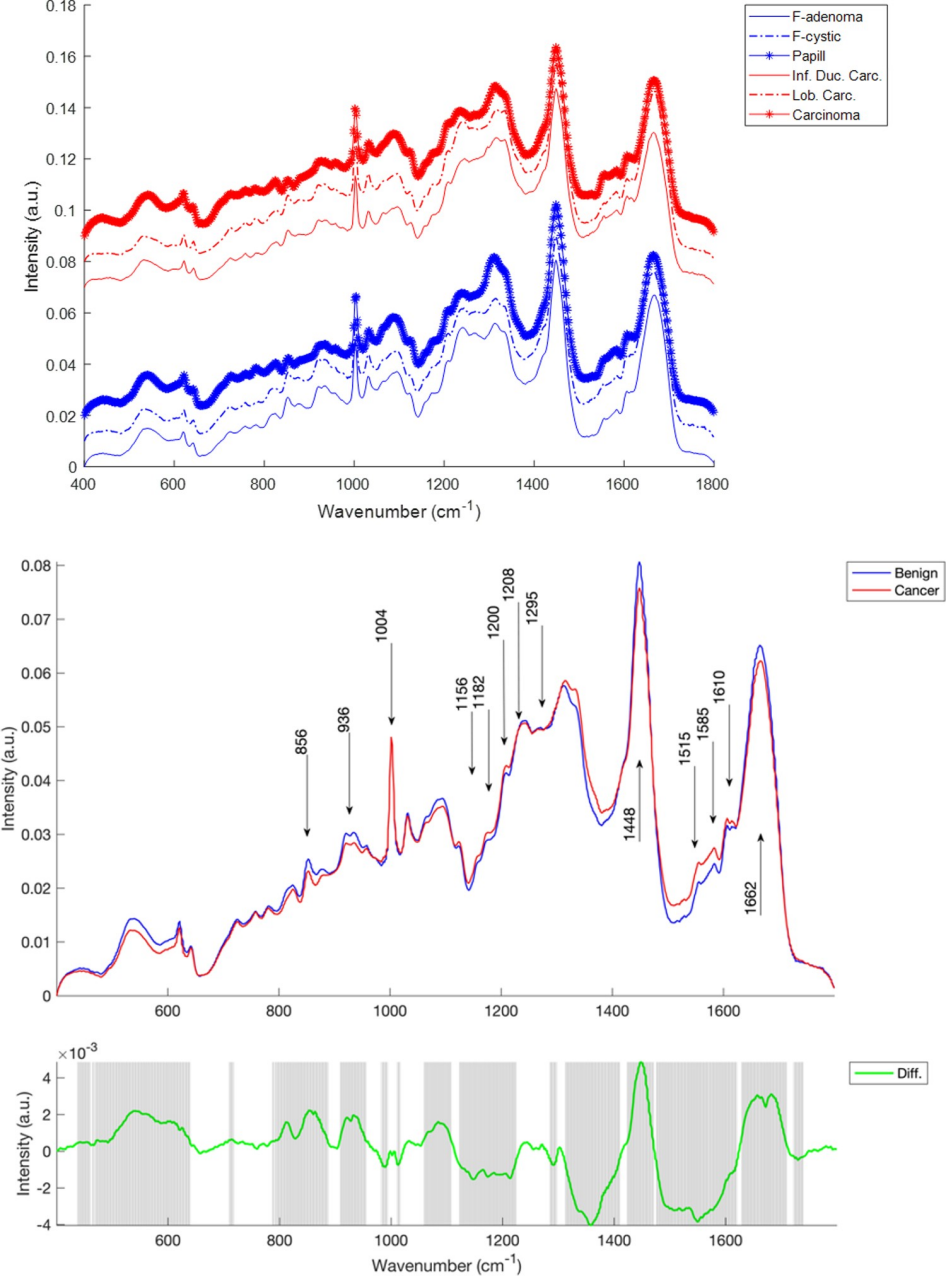
Top panel, mean spectra by subclass, with benign tissues (fibrocystic adenoma, fibrocystic disease and intraductal papilloma) in blue and cancerous tissues (infiltrating ductal carcinoma, infiltrative lobular carcinoma and carcinoma) in red. Spectra are offset for clarity. Middle panel, mean spectra of benign (blue) and cancerous tissue (red), Bottom panel, difference spectrum (green) and shaded regions in which a 2-tailed t-test has identified differences that are significant at the level of p<1×10^−4^.

Fig 3 shows mean Raman spectra by subclass (fibrocystic lesion, fibroadenoma, intraductal papilloma, infiltrative ductal carcinoma and lobular carcinoma) and by class (benign and cancer). In each the dominant features differentiating the classes as shown in the difference spectrum (Fig 3 bottom panel) include vibrations in the region from 800–985 cm^-1^ and from 1120–1690 cm^-1^. Some important vibrational modes within these and other regions of the spectrum are labelled in Fig 3.

A series of classifiers were created, including PCA-LDA, PCA-QDA, PLSDA, Linear c-SVC, Linear nu-SVC, RBF c-SVC and RBF nu-SVC, which were used to determine whether Raman spectroscopy could discriminate between benign lesions and cancer.

**Fig 4:**
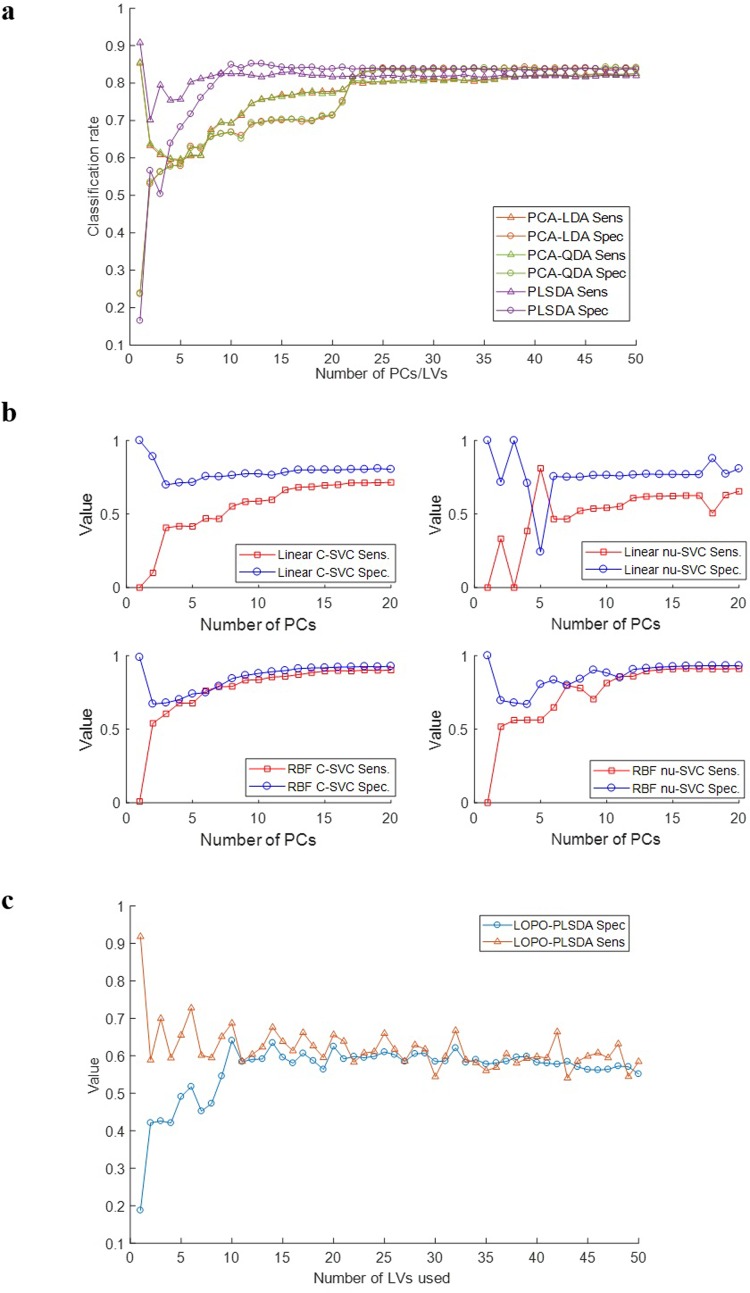
a) Sensitivity and specificity with respect to number of PCs/ LVs for various models in classifying benign lesions versus cancer. b) Sensitivity and specificity with respect to number of PCs for various models in classifying benign lesions versus cancer. c) Sensitivity and specificity with respect to number of LVs for PLSDA model in classifying benign lesions versus cancer using LOPOCV.

Fig 4A shows the sensitivity and specificity for PCA-LDA, PCA-QDA and PLSDA models. For PCA-LDA and PCA-QDA, improved sensitivity and specificity could be achieved with PC scores >22. PCA-LDA and PCA-QDA gave similar results across the number of PC scores used. The maximum sensitivity and specificity achieved was approx. 83% and 80% respectively. For PLSDA, the optimal result was obtained using 10–17 LVs, resulting in sensitivity and specificity of 82% and 84% respectively.

Fig 4B shows the sensitivity and specificity for Linear c-svc, Linear nu-svc, RBF c-svc and RBF nu-svc SVM models. For Linear c-svc and nu-svc SVM models, the optimal result was obtained from 12 PC scores, resulting in sensitivity and specificity of <71% and <80% respectively. For RBF c-svc SVM, the optimal result was obtained from 15 PC scores with 90% sensitivity and <92% specificity while for RBF nu-svc SVM, the optimal result was obtained from 15 PC scores with ~90% sensitivity and ~93% specificity.

Fig 4C shows the sensitivity and specificity for the PLSDA model using LOPOCV. The optimal result was obtained from 10 LV scores, resulting in ~64% sensitivity and ~68% specificity.

## Discussion

### Immunohistochemistry

Immunohistochemical staining for ER and HER2/neu has been performed on the five types of breast tissues shown in Fig 2. H&E images of benign breast conditions and benign and malignant tumors are presented in Fig 1. Benign breast lesions are not cancerous since they do not spread to other locations in the body. The benign conditions diagnosed in this study are fibrocystic, fibroadenoma and intraductal papilloma.

Fibrocystic disease of the breast can have different patterns, proliferative, that might be associated with an increased risk of breast carcinoma, and non- proliferative. Some of these alterations like stromal fibrosis and macro-cysts produce palpable “lumps”. Non proliferative change is the most common type of alteration, characterized by an increase in fibrous stroma associated with dilation of ducts and formation of cysts of various sizes [22]. The relationship of fibrocystic changes to breast carcinoma is a medically controversial area. Fig 1 shows the dilated ducts, cysts formation with sclerosing adenosis. ER staining shows focal positivity in fibrocystic disease (Fig 2) and HER2/neu shows negativity (Fig 2).

Fibroadenoma is a benign tumour of the breast with overgrowth of both glandular and fibrous tissue. It has two common histological patterns pericanalicular and intracanalicular. *Pericanalicular* has open glandular spaces and *intracanalicular* has compressed glandular spaces but there is no clinical significance to this distinction. The fibroadenoma section in Fig 1B is of pericanalicular type and shows focal ER positivity (Fig 2) and negative HER2/neu (Fig 2).

Another type of benign tumor is intraductal papilloma which is composed of proliferative epithelium but with myoepithelial cell lining and branching arbor of fibrovascular cores. The lesion is usually found in the large distal ducts and can become fibrotic or calcifies with age [22]. The intraductal papilloma case mentioned in the result (Fig 2) shows clear dilated ducts with papillary projections having fibrovascular core and epithelial myoepithelial lining. The ER staining shows patchy positivity (Fig 2) and HER2/neu is negative in intraductal papilloma (Fig 2).

Invasive Ductal Carcinoma (IDC) is the most common type of breast carcinoma. The tumor arises from the terminal duct lobular unit. The tumor is characterized by the tendency of cells for tubular formation associated with desmoplastic reaction (fibrosis).

The section through the tumor (Fig 1) shows proliferating neoplastic cells, some forming tubules with moderate desmoplastic reaction and mild lymphocytic infiltration. ER (Fig 2) is strongly diffusely positive and HER2/neu is positive in invasive ductal carcinoma (Fig 2).

Infiltrative lobular carcinoma (ILC) consists of cells that invade individually into stroma and are often aligned in strands or chains. The classical form of ILC is characterized by small relatively uniform neoplastic cells that invade the stroma singly and in a “single-file” pattern resulting in linear strands [22]. The cells may encircle the mammary ducts and infiltrate the stroma and adipose tissue without desmoplastic reaction. ER shows strongly diffusive positivity (Fig 2) and HER2/neu is positive in infiltrative lobular carcinoma tissues (Fig 2).

### Raman spectral comparison

The fingerprint region contains diagnostic bands from proteins, lipids, nucleic acids and other biomolecules including carotenoids and calcium hydroxyapatite. Additional bands assigned to amino acids are observed in this region including: tyrosine, phenylalanine, tryptophan, proline and valine.

In Fig 3 several modes of vibration were found to be significantly different between the benign and cancer classes. The band at 1662 cm^-1^ is assigned to the amide I mode originating mainly from proteins and nucleic acids. The two weak bands at 1610 and 1585 cm^-1^ observed in the breast tissue are due to the ν(C = C) modes of aromatic amino acids (phenylalanine, tyrosine, and tryptophan). The band at 1448 cm^-1^ is assigned to the ν(CH_2_/CH_3_) modes from a combination of lipo-proteins from the cell membrane, adipose tissue, and nucleic acids. The amide III bands are observed in the region of 1295–1200 cm^-1^, which are attributed to a combination of ν(CN) and ν(NH) modes of the peptide bond ν(-CONH). Other bands assigned to the amino acids include: the ν(C-C) modes of tryptophan and phenylalanine at 1208 cm^-1^, the ν(G-H) mode of tyrosine at 1182 cm^-1^, the ν(C-N), ν(G-H) and ν(C-C) ring breathing modes of phenylalanine at 1153, 1026 and 1002 cm^-1^, respectively. The bands at 936 and 856 cm^-1^ are assigned to the ν(C-C) modes of proline and valine, and the ν(C-CH) modes of proline and tyrosine, respectively. The spectrum exhibits three major characteristic bands in this region including those due to: the ν(C = C) mode at 1515 cm^-1^, the ν(C-C) mode at 1156 cm^-1^, and the ring breathing mode at 1004 cm^-1^.

### Discrimination of Raman spectra with PCA analysis

Principal component analyses were applied to all of the Raman spectra collected from breast tissues that had been subjected to the same sampling preparation and presentation techniques (sectioning and sample mounting). The analysis aimed to model the data with the least number of principal components. Ideally the most parsimonious model which results in the highest degree of predictive accuracy is desirable in this instance. S1 Fig (supplementary information) demonstrates the high degree of overlap between spectral features in the cancer and benign classes. As such it is difficult to develop models that spectrally discriminate between data in the two classes using two to three data dimensions, which then would allow visualisation of the principal component scores here. Therefore the performance of high-dimensional classification algorithms has been examined as the dimensionality of the data is increased (Fig 4).

The performance of the different algorithms, PCA-LDA, PCA-QDA, PLSDA, Linear c-SVC, Linear nu-SVC, RBF c-SVC and RBF nu-SVC, were evaluated using sensitivity and specificity. Sensitivity and specificity are common statistical measures quantifying the degree to which a test correctly identifies a positive and negative medical condition, respectively. Sensitivity is a measure of the ability of the test to identify positive cases while specificity is a measure of the ability of the test to identify negative cases, both within mixed populations of positive and negative cases. Sensitivity and specificity were calculated based on the results from the Raman data and from the gold standard histopathology.

The samples were divided into two classes: class 1 having benign lesion samples and class 2 having cancer samples. This data set was created to evaluate the power of the algorithms to differentiate cancer samples from benign lesions. Each classifier was trained using 60% of the spectra randomly selected from all spectra, and tested using the remaining held out 40%. Applying PCA-LDA and PCA-QDA to the data, classification improved for PC scores > 22, as shown in Fig 4A. For classification purposes, the PCA-LDA and PCA-QDA models had very similar performances, with sensitivity and specificity >80%. Applying PLSDA to the data, classification improved for 10–17 LVs and sensitivity and specificity of 82% and 84% respectively was achieved as shown in Fig 4. A number of SVM models were also tested. For Linear c-svc and nu-svc SVM models, classification improved for PC scores > 12 and sensitivity and specificity of <71% of and <80% respectively was achieved as shown in Fig 4B. For RBF c-svc and nu-svc SVM, classification improved for PC scores > 15 and 90% sensitivity and >92% specificity was achieved as shown in Fig 4B. The RBF SVM models were found to perform best for classification of benign lesions and cancer but required more processing time than PCA LDA, PCA QDA and PLSDA.

PLSDA was also carried out with leave one patient out cross validation (LOPOCV) as shown in Fig 4C. Classification improved for LV scores >10 but relatively poor sensitivity of 64% and specificity of 68% was achieved. This was most likely due to the low number of patient samples and further work would be required to increase the sample size.

Overall, good classification of benign lesions and cancer cases could be achieved particularly with the RBF SVM models. The combination of Raman analysis and these types of chemometric techniques can provide very acceptable findings for developing fast, accurate, less-invasive, and non-analysis dependent clinical procedures, especially for screening purposes.

## Conclusions

In conclusion, this study shows the ability of Raman spectroscopy to discriminate between benign breast lesions (fibrocystic, fibroadenoma, intraductal papilloma) and breast cancer (invasive ductal carcinoma and lobular carcinoma). The benign and cancer cases could be classified with good sensitivity and specificity using a number of different models. PCA-LDA, PCA-QDA and PLSDA models achieved similar sensitivity and specificity of >80%. RBF SVM models achieved sensitivity and specificity of >90% but required more processing time than the PCA-LDA, PCA-QDA and PLSDA models. Further studies are required to increase the sample size and to validate the findings using an independent cohort of patients.

## Supporting information

S1 Fig **Principal component scores plots for tissue classed as either (a) infiltrating ductal or lobular carcinoma (LC) (black) or (b) fibrocystic lesion, fibroadenoma or intraductal papilloma (red)**.
(TIF)
