# Correction: Discrimination of breast cancer from benign tumours using Raman spectroscopy

**DOI:** 10.1371/journal.pone.0216311

**Published:** 2019-04-25

**Authors:** Fiona M. Lyng, Damien Traynor, Thi Nguyet Que Nguyen, Aidan D. Meade, Fazle Rakib, Rafif Al-Saady, Erik Goormaghtigh, Khalid Al-Saad, Mohamed H. Ali

The following information is missing from the Funding section: This article was funded by the Qatar National Library. The funder had no role in study design, data collection and analysis, decision to publish, or preparation of the manuscript.
