# Supplementary figures and images for: Discrimination of breast cancer from benign tumours using Raman spectroscopy
Source: PLoS One. 2019 Feb 14;14(2):e0212376. doi: 10.1371/journal.pone.0212376 (PMC6375635; doi:10.1371/journal.pone.0212376)

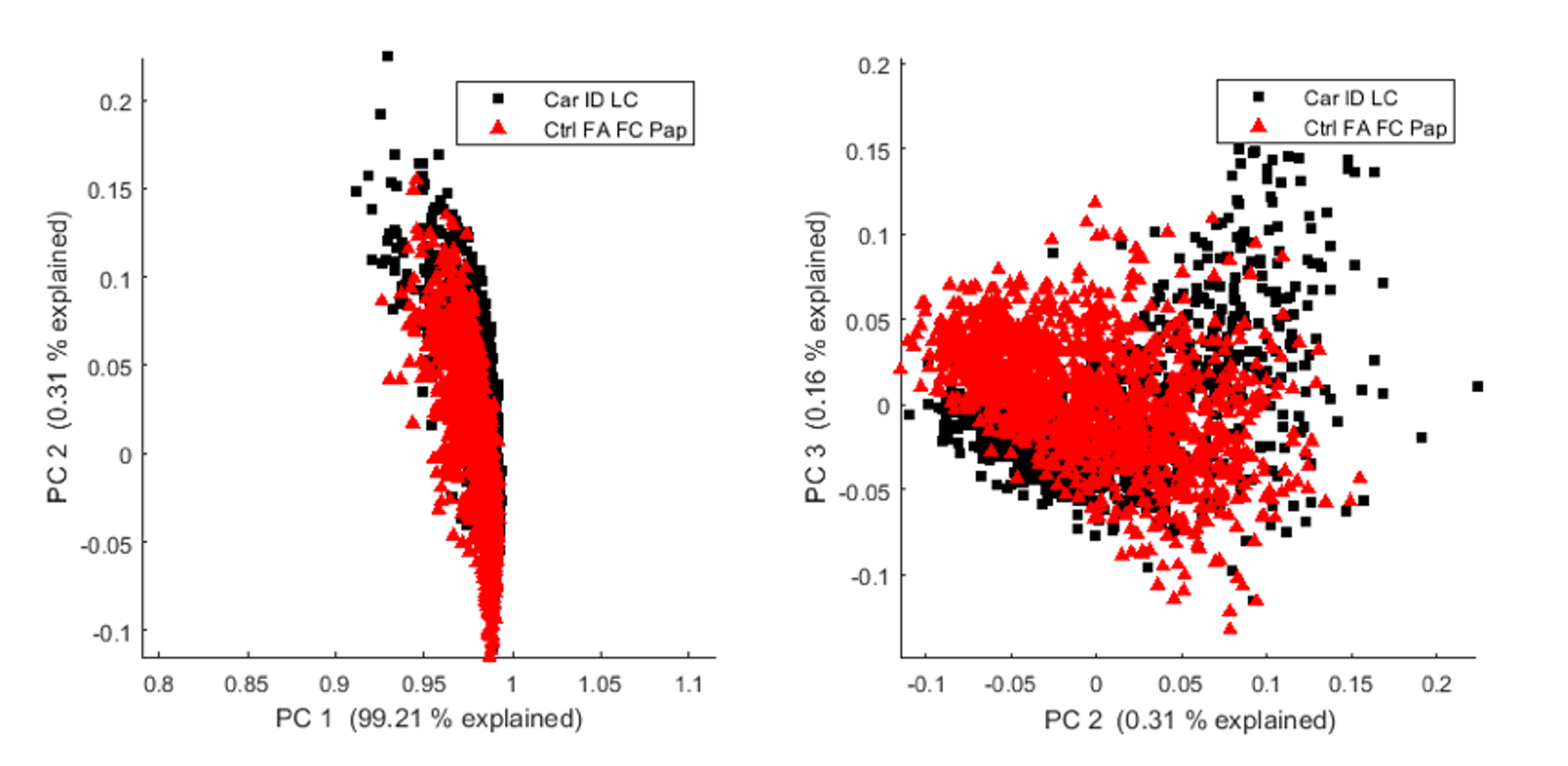

Supplement: S1 Fig — Principal component scores plots for tissue classed as either (a) infiltrating ductal or lobular carcinoma (LC) (black) or (b) fibrocystic lesion, fibroadenoma or intraductal papilloma (red). (TIF) [file pone.0212376.s001.tif]
